# Studies of lipopolysaccharide effects on the induction of α-synuclein pathology by exogenous fibrils in transgenic mice

**DOI:** 10.1186/s13024-015-0029-4

**Published:** 2015-07-30

**Authors:** Nicola J. Rutherford, Amanda N. Sacino, Mieu Brooks, Carolina Ceballos-Diaz, Thomas B. Ladd, Jasie K. Howard, Todd E. Golde, Benoit I. Giasson

**Affiliations:** Center for Translational Research in Neurodegenerative Disease, Department of Neuroscience, University of Florida, 1275 Center Drive, Room BMS J-483, PO Box 100159, Gainesville, FL 32610 USA

**Keywords:** Lipopolysaccharide, α-synuclein pathology, Transgenic mice, Neuroinflammation, Parkinson’s disease

## Abstract

**Background:**

Parkinson’s disease (PD) is a progressive neurodegenerative disorder that is pathologically characterized by loss of dopaminergic neurons from the substantia nigra, the presence of aggregated α-synuclein (αS) and evidence of neuroinflammation. Experimental studies have shown that the cerebral injection of recombinant fibrillar αS, especially in αS transgenic mouse models, can induce the formation and spread of αS inclusion pathology. However, studies reporting this phenomenon did not consider the presence of lipopolysaccharide (LPS) in the injected αS, produced in *E. coli*, as a potential confound. The objectives of this study are to develop a method to remove the LPS contamination and investigate the differences in pathologies induced by αS containing LPS or αS highly purified of LPS.

**Results and conclusions:**

We were able to remove >99.5 % of the LPS contamination from the αS preparations through the addition of a cation exchange step during purification. The αS pathology induced by injection of fibrils produced from αS containing LPS or purified of LPS, showed a similar distribution pattern; however, there was less spread into the cortex of the mice injected with αS containing higher levels of LPS. As previously reported, injection of αS fibrils could induce astrogliosis, and αS inclusions were present within astrocytes in mice injected with fibrils comprised of αS with or without cation exchange purification. Furthermore, we identified the presence of αS pathology in ependymal cells in both groups of mice, which suggests the involvement of a novel mechanism for spread in this model of αS pathology.

**Electronic supplementary material:**

The online version of this article (doi:10.1186/s13024-015-0029-4) contains supplementary material, which is available to authorized users.

## Background

Parkinson’s disease (PD) is characterized by the profound loss of nigral dopaminergic neurons, the presence of proteinacious inclusions comprised of aggregated α-synuclein (αS) in some of the remaining neurons, and neuroinflammation in affected brain regions [1–4]. PD is related to several other neurodegenerative diseases, including dementia with Lewy bodies (DLB), due to the presence of pathological αS inclusions [5–7]. Studies of human pathology have supported the notion that αS aggregation may spread along neuroanatomical pathways associated with disease progression [8, 9].

It is believed that one method involved in the spread of αS pathology associated with disease progression is cell-to-cell transmission of aggregated αS seeds followed by prion-like conformational templating. This notion has been supported by the presence of αS inclusions within fetal dopaminergic neuronal transplants in the brains of PD patients [10–12]. Experimental mouse studies using recombinant fibrillar αS, produced in bacteria, to induce the spread of αS pathology throughout the neuroaxis of αS transgenic (Tg) and non-Tg mice have further suggested that αS aggregation may be able to spread by “prion-like” conformational templating mechanisms [13–18]. However, most of these studies did not take into account that the possible presence of bacterial endotoxin/lipopolysaccharide (LPS) may confound some of these results. The importance of this issue is underscored by reports that the intracerebral or peripheral administration of LPS alone to mice Tg for αS containing the PD-causing A53T mutation (M83 line), which were used to show cerebral spread of αS inclusion pathology using bacterial recombinant αS aggregates [13, 16], can induce CNS αS inclusion pathology [19, 20]. LPS is a major component of the *E. coli* bacterial cell wall and is a potent inducer of inflammation, by activating the toll-like receptor 4 (TLR4) [21, 22], nevertheless, endogenous αS itself can reportedly trigger an immune response through a similar mechanism [23–27].

Activation of the innate neural immune response is a consistent finding associated with PD and it is believed to contribute to disease pathogenesis [3, 28, 29], however it is unknown whether this process is responsible for the onset of disease, it’s progression and/or if it exacerbates existing neurodegeneration. Neuroinflammation is evidenced by the presence of astrogliosis, activated microglia (the immune cells of the CNS) and increased levels of proinflammatory cytokines [30–32]. Furthermore, it is believed that a chronic brain inflammatory state can be damaging, as factors produced and released during such a state can cause/add to oxidative stress [32].

In this study we show that αS purified from *E. coli* consistently contains LPS that is difficult to completely remove, likely due the lipid binding and ionic properties of αS [33]. Nevertheless, performing stereotaxic brain injections with preformed fibrils comprised of αS highly purified of LPS compared to αS with co-purified LPS in αS Tg mice, we demonstrate similar induction and spread of αS throughout the neuroaxis. Additionally, we show that the removal of LPS from the injected αS does not affect the number of GFAP-reactive astrocytes present in the hippocampus or entorhinal cortex, and αS inclusions are present in astrocytes in both injection groups. Interestingly, during the course of these studies, we observed that in this induced model of αS inclusion pathology there is not only significant induction of aggregates in neurons and glia, but also in ependymal cells. This expands the number of cell types that can contain αS pathology and could have important implications for the mechanism involved in the spread of αS inclusion pathology.

## Results

### Quantification of endotoxin/LPS contamination in standard αS protein preparations

**Fig. 1:**
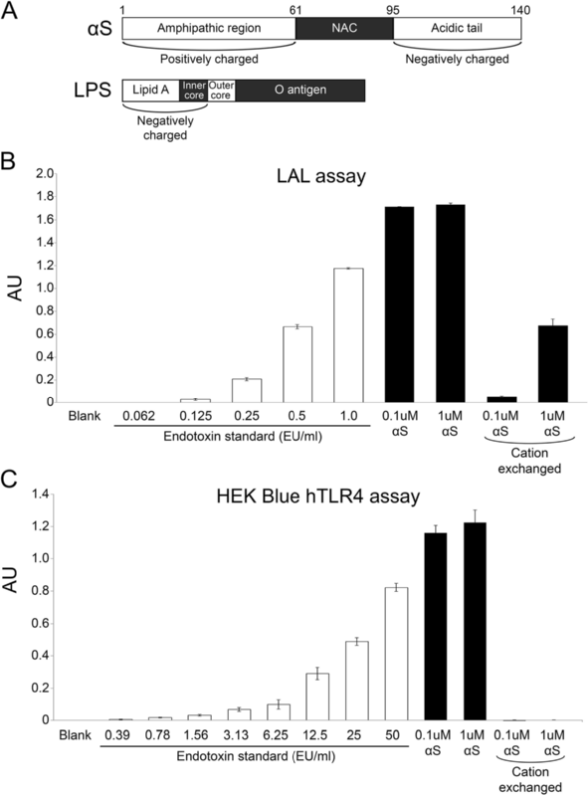
Assessment of endotoxin/LPS contamination in αS protein preparations. **a** Schematics showing the charged regions and hydrophobic regions of αS and LPS. NAC = “non-amyloid-β component of the amyloid plaque” hydrophobic region of αS. (**b** and **c**) Average quantification of endotoxin contaminants in αS protein preparations. Results are also shown for samples that were further purified using High S resin (cation exchanged). (b) and (c) illustrate contamination in the same sample set, using the Pierce LAL assay and the InvivoGen HEK-Blue-hTLR4 cell culture system respectively. The hTLR4 assay shows an expanded standard curve. 1 μM αS = 0.0146 mg/ml. White bars represent the endotoxin standard and black bars represent test samples. Error bars represent the standard error of the mean. AU = absorbance units

αS protein was expressed in and purified from *E. coli*, which natively contains endotoxins, i.e. LPS. LPS is an abundant molecule within the membrane of Gram-negative bacteria, and is a notorious contaminant of protein purified from *E. coli* [34, 35]. LPS is a potent activator of the immune response; injection of just 4 ng/kg body weight (approximately 40EU/kg) can cause a dramatic increase in inflammatory markers in humans [36]. The United States Pharmacopeia (USP) recommends that vaccines/injectable medications contain <5EU/kg body weight [37]. It is also well known that it is difficult to completely remove LPS from bacterial protein preparations due to its ionic and hydrophobic properties, and the formation of a spectrum of multimeric complexes with varied size properties [34, 38, 39]. As αS is a lipid binding protein [33] and it has hydrophobic, negatively charged and positively charged domains (Fig. 1a), we wanted to assess the extent to which our standard bacterial protein preparations may be contaminated with LPS. We employed the standard LAL *in vitro* method and a TLR4 cell responder assay to determine the levels of endotoxin contamination, using purified endotoxin as our standard (Fig. 1b and c). We found that our bacterial αS protein preparations contained varying levels of LPS, as shown for one preparation in Fig. 1. The TLR4 assay indicates that there are >50EU/ml in 0.1 μM protein sample (i.e. >34.6 EU/μg αS protein). Trying different methods, we found that it was difficult to remove the LPS from our preparations, but by lowering the pH to 4.2 and using cation affinity purification with extensive washing, we were able to remove >99.5 % of the bacterial endotoxin contamination from our recombinant αS protein samples, although some residual amounts (<0.5EU/ml in 1 μM protein sample; <0.035 EU/μg αS protein) were still detected with the more-sensitive LAL assay (Fig. 1c). Samples from these same preparations of recombinant human αS prior to cation exchange purification (αS) or after purification (cation exchanged αS) were fibrillized, sonicated and used for stereotaxic injection into αS Tg mice. Following surgery, the LAL assay was repeated on the remaining αS fibrils. We found that although the amount of endotoxin within the cation exchanged αS fibrils increased (~5EU/ml in 1 μM protein sample; ~0.35EU/μg αS protein), there was ≥99 % less endotoxin present in these αS fibrils than in the non-cation exchanged αS fibrils.

### Effects of endotoxin contamination on the induction and spread of αS pathology in M83 αS Tg mice

**Fig. 2:**
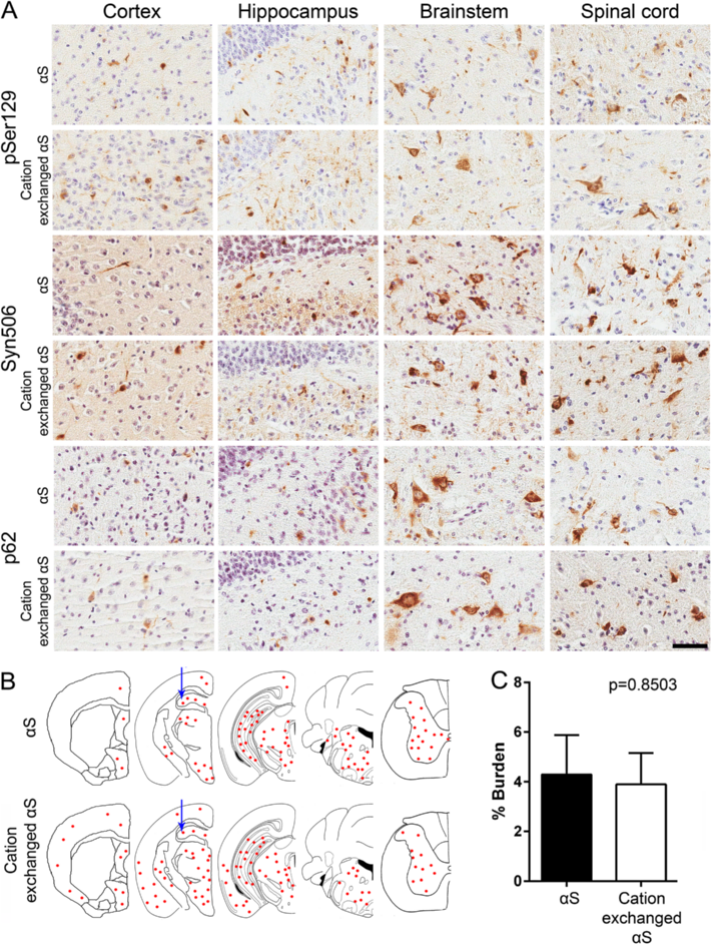
αS pathology in M83 αS Tg mice following injection of αS or cation exchanged αS fibrils. **a** Representative immunohistochemical images of cortical, hippocampal, brainstem, and spinal cord sections of hemizygous M83 αS Tg mice stereotaxically injected in the hippocampus with αS or LPS purified (cation exchanged) αS. Antibodies to αS (Syn506), to αS phosphorylated at Ser129 (pSer129/81A), and a general inclusion marker (p62) were used. Scale bar = 50 μm. **b** Schematic representation of the distribution of αS inclusion pathology in the neuroaxis of hemizygous M83 αS Tg mice injected with αS or cation exchanged αS fibrils. Red dots indicate the sites and amounts of αS pathology. Blue arrows indicate the site of injection. **c** Quantification of Syn506 staining in the hippocampus of mice injected with αS or cation exchanged αS fibrils. The p-value was calculated using a two-tailed *t*-test. Error bars represent the standard error of the mean

**Fig. 3:**
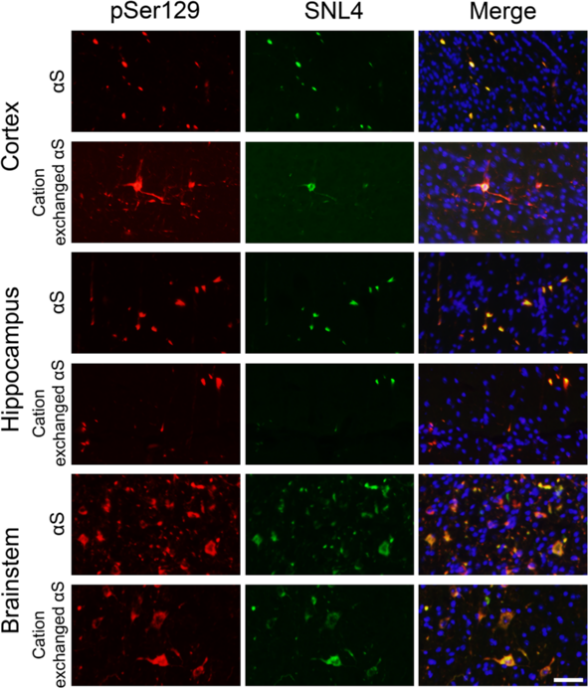
Immunofluorescence analysis of αS pathology in M83 αS Tg mice following injection of αS fibrils. Representative immunofluorescent images of the cortical, hippocampal, and brainstem regions of hemizygous M83 αS Tg mice injected with αS or cation exchanged αS fibrils, stained with antibodies to phosphorylated αS (pSer129/81A; red) or αS (SNL4; green) and DAPI (blue). Individual images were overlaid to show colocalization (Merge). Scale bar = 50 μm

M83 αS Tg mice express human A53T αS driven by the mouse prion-protein promoter. These mice develop an age-dependent motor phenotype resulting in paralysis that is associated with the formation of αS inclusions in the spinal cord, brain stem and midbrain [40]. These pathological changes can occur between 7 and 16 months of age in homozygous M83 αS Tg mice, but later than 22 months of age in hemizygous mice [40]. Intracerebral injections of preformed αS amyloid fibrils, generated from recombinant αS produced in *E. coli,* have been shown to induce the formation of αS inclusion pathology that spreads from the site of injection [13, 15, 16]. However, levels of LPS contamination were not taken into consideration in these studies, and it was previously reported that both peripheral or brain injection of LPS can induce αS inclusion pathology in M83 αS Tg mice [19, 20]. Given that we were able to generate recombinant αS that is largely devoid of LPS, we tested whether LPS contamination significantly altered the induction of brain αS inclusion pathology following intracerebral injection of preformed amyloid fibrils. Fibrils produced from αS and cation exchanged αS were stereotaxically injected into the hippocampus of 2 month old hemizygous M83 αS Tg mice. Immunohistochemical analysis of the brains and spinal cords of these mice (Fig. 2a) revealed the presence of inclusions that were immunoreactive for antibodies to αS (Syn506), Ser129 phosphorylated αS (pSer129/81A) and a general inclusion marker (p62) in mice injected with fibrils produced from αS or cation exchanged αS. Formation of authentic αS inclusion pathology was also confirmed by double-immunofluorescence microscopy with antibodies to αS (SNL4) and Ser129 phosphorylated αS (pSer129/81A; Fig. 3). Distribution analysis of the αS pathology (Fig. 2b) showed a high concentration of inclusions at the site of injection in both sets of mice and quantification of Syn506 staining in this region revealed no difference between the two groups (Fig. 2c). Pathology was observed to have spread caudally, to the brainstem and spinal cord in both groups, with similar frequencies of inclusions. The only major difference detected between the distributions of inclusions in the two groups was the spread of αS pathology into the cortex of the mice. In mice injected with cation exchanged αS, inclusions appeared with moderate frequency throughout the cortex, whereas cortical inclusions in the αS injected mice were rare, and only observed in dorsal regions.

**Fig. 4:**
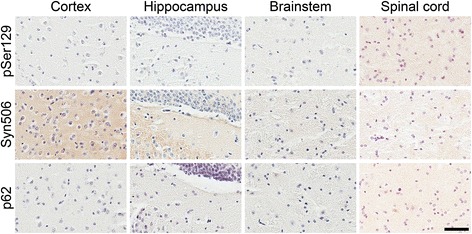
Immunohistochemical analysis showing the paucity of αS pathology in M83 αS Tg mice injected with LPS. Representative immunohistochemical images from the cortex, hippocampus, brainstem, and spinal cord of homozygous M83 αS Tg mice injected with LPS, using antibodies to αS (Syn506), to αS phosphorylated at Ser129 (pSer129/81A), and a general inclusion marker (p62). No immunoreactive inclusions were identified in these mice. Scale bar = 50 μm

To further assess the involvement of LPS contamination in driving the formation of αS pathology, we stereotaxically injected homozygous M83 αS Tg mice in the hippocampus with 10 μg of purified LPS, with an activity of ~0.25 EU/pg (Additional file 1: Figure S1A). This constitutes greater than 10 million fold more endotoxin units than were present in the cation exchanged αS used for injection. We used homozygous mice as they should be more primed for the induction of pathology, but in these studies no αS inclusion pathology was observed (Fig. 4). Similarly, injection of PBS into the hippocampus of homozygous M83 αS Tg mice did not induce the formation of αS inclusion pathology (data not shown) indicating that it is the injected αS fibrils that are responsible for the induction of αS inclusions. We confirmed that the purified LPS was able to induce an inflammatory response, by treating primary microglial cultures produced from nTg mice with the same LPS that was injected. As expected, we observed changes in morphology of the cells over time; they became round and swollen with vacuoles evident after 12 h of treatment with LPS (Additional file 1: Figure S1B). We also detected a concomitant rise in protein levels of interleukin-6 (IL-6), a marker of inflammation, within the media of the LPS treated cells after 6 h of treatment, which increased to 19.6 ng/ml compared to media from control cells, which remained below the level of detection (Additional file 1: Figure S1C).

### Astrocyte activation and induction of astrocytic αS inclusion pathology due to hippocampal injection of recombinant of αS fibrils

**Fig. 5:**
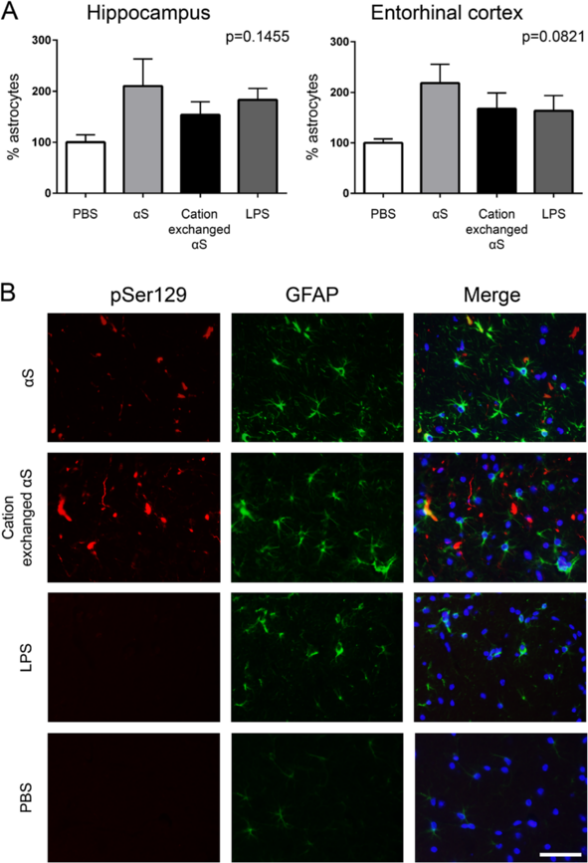
Astrogliosis and αS inclusions within astrocytes, in M83 αS Tg mice injected with αS fibrils. **a** Percentage of GFAP-positive astrocytes per field area in the hippocampus (left) and entorhinal cortex (right) of M83 αS Tg mice stereotaxically injected with αS or cation exchanged αS fibrils or LPS in the hippocampus, versus PBS injected mice. P-values were determined by one-way ANOVA analyses. **b** Representative images of the hippocampus, showing double immunofluorescent staining with pSer129/81A (αS phosphorylated at Ser129) and GFAP. Individual images were overlaid (Merge) to show colocalization. Scale bar = 50 μm

We recently reported that the hippocampal injection of fibrillar αS in M83 αS Tg mice also resulted in the induction of astrogliosis and that a significant proportion of induced αS inclusion pathology was actually in glial cells [41]. To assess if the changes in glia were due to the presence of LPS in the fibrillar αS that was injected, we assessed the abundance of astrogliosis, by staining for GFAP, in the brains of M83 αS Tg mice that were injected with αS, cation exchanged αS, LPS or PBS. In this cohort of mice, the density of GFAP-positive astrocytes in the hippocampus and entorhinal cortex was elevated in αS and LPS injected mice compared to PBS controls, but did not reach statistical significance (Fig. 5a). We also assessed whether there was similar induction of αS inclusion pathology in glial cells and found that αS inclusions were present in astrocytes in both αS and cation exchanged αS injected mice (Fig. 5b).

### Induction of ependymal αS inclusion pathology resulting from the hippocampal injection of recombinant αS fibrils

**Fig. 6:**
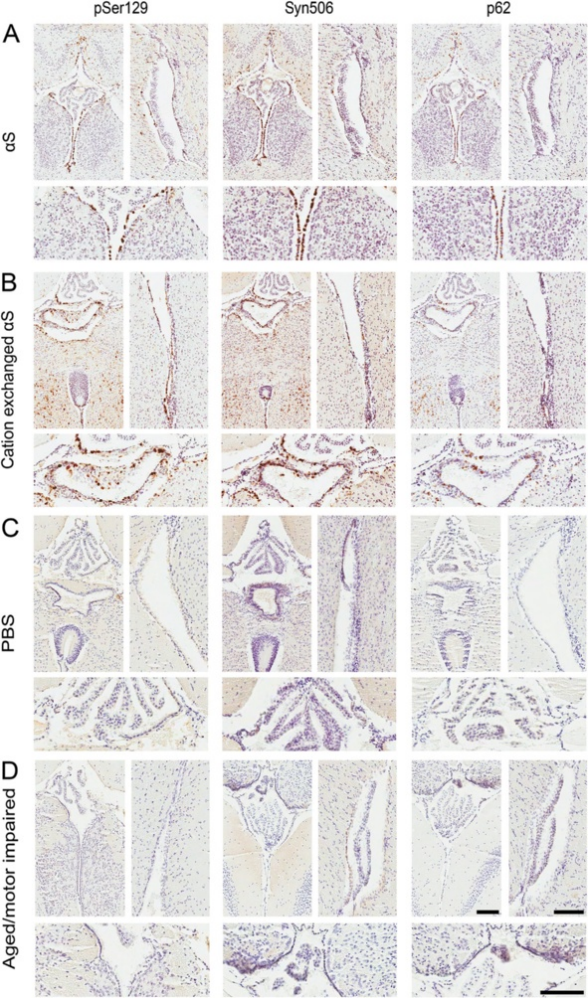
Immunohistochemical analysis showing ependymal cell αS pathology in M83 αS Tg mice injected with αS fibrils. Representative immunohistochemical images of hemizygous M83 αS Tg mice injected with αS fibrils (**a**) or cation exchanged αS fibrils (**b**), and homozygous M83 αS Tg mice injected with PBS (**c**), or aged until motor impairments were observed (**d**). Images showing midline (left; below showing higher magnification) and lateral ventricle (right) stained with antibodies to αS phosphorylated at Ser129 (pSer129/81A), αS (Syn506) and a general inclusion marker (p62). Scale bars = 100 μm

**Fig. 7:**
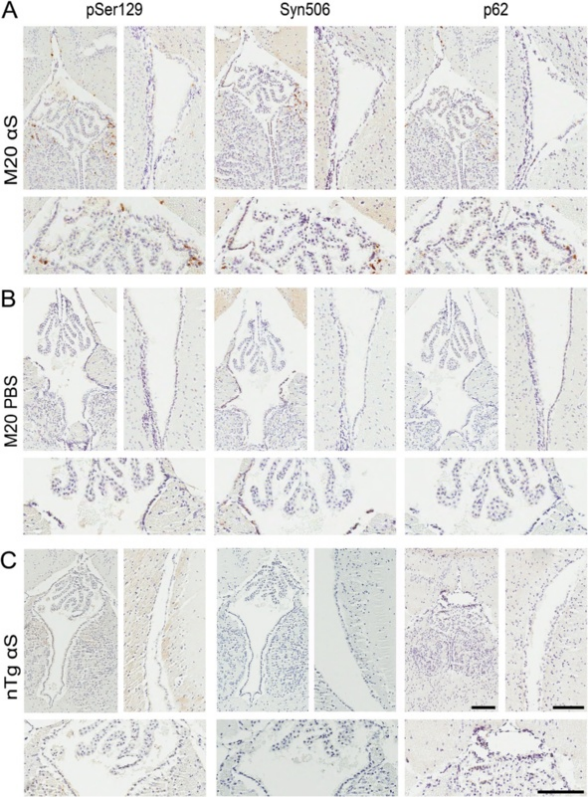
Immunohistochemical analysis showing ependymal cell αS pathology in M20 αS Tg mice injected with αS fibrils. Representative immunohistochemical images of hemizygous M20 αS Tg mice injected with αS fibrils (**a**) or PBS (**b**) and nTg mice injected with αS fibrils (**c**). Images showing midline (left; below is a higher magnification) and lateral ventricle (right) stained with antibodies to αS phosphorylated at Ser129 (pSer129/81A), αS (Syn506) and a general inclusion marker (p62). Scale bars = 100 μm

Expending the observations of αS inclusion pathology in glia, we also observed a similar induction of αS inclusions in ependymal cells of M83 αS Tg mice injected in the hippocampus with preformed αS fibrils (Fig. 6a and b). In comparison, αS inclusion pathology within ependymal cells was not observed in naïve M83 αS Tg mice that become motor impaired and develop αS inclusions due to aging (Fig. 6d). To assess if the formation of ependymal αS pathology in αS injected M83 αS Tg mice was unique to this mouse line, we re-examined the pathology in M20 αS Tg mice that were also challenged with hippocampal injection of fibrillar αS (Fig. 7a). We found that ependymal αS inclusion pathology was also present in these mice, albeit less abundant than in M83 αS Tg mice. We did not detect any ependymal cell αS pathology in either mouse line injected with PBS (Figs. 6c and 7b). Furthermore, injection of αS fibrils into non-Tg mice did not induce the formation of αS inclusion pathology within ependymal cells (Fig. 7c).

## Discussion

In this study we showed that recombinant αS prepared from *E. coli* can readily contain bacterial endotoxin/LPS, which most likely occurs due to the complex electrostatic, hydrophobic, and multimeric structure properties of both αS and LPS [34, 38, 39, 42]. The identification of LPS contamination in these protein preparations is an important finding since recombinant αS has been injected into αS Tg mice, in the form of fibrillar seeds, in order to induce pathology [13, 15–18]. Since LPS is a potent inflammagen, and chronic neuroinflammation is believed to play a substantial role in the development and/or progression of a number of neurodegenerative diseases, including PD [6, 28, 29, 43, 44], it stands to reason that its presence would be a significant confound. Indeed it has been published that a single injection of LPS in the brain or peripherally is sufficient, in M83 αS Tg mice, to induce αS inclusion pathology [19, 20]. To assess for the involvement of the contaminating LPS in the induction of αS pathology in these mice, we developed a purification protocol that eliminates almost all of the LPS contaminants (>99.5 %), by exploiting the presence of a region of positive charge that is present in αS, but not in LPS. Stereotaxic hippocampal injections in M83 αS Tg mice, using fibrils prepared from αS with or without the additional purification (cation exchanged αS and αS, respectively), induced similar amounts and distributions of αS inclusion pathology except in the cortex, where we observed fewer αS inclusions in the αS injected mice. Moreover, we were unable to reproduce the results published by Gao and colleagues [19, 20]; we observed no αS inclusion pathology in homozygous M83 αS Tg mice injected with purified LPS. However, there were major differences between these studies. First, we injected into the hippocampus whereas Gao et al. injected LPS into the substantia nigra. The dopaminergic cells of the substantia nigra are particularly vulnerable to inflammatory/oxidative stress that can be induced by treatment with LPS, and so the overexpressed αS may be induced to aggregate due to additional adverse cellular mechanisms. Second, the age at which the injections were carried out was different between the two studies. Our mice were injected at 2 months of age, an age which is much earlier than when naïve mice develop pathology or motor symptoms. Gao et al. injected their mice at 7 or 12 months of age, for intraperitoneal and brain injection respectively, which is around the age at which these mice typically develop pathology. They reported that the mice did not present with an overt neurological phenotype at the time of injection however, the mice could already have αS pathology within their brains or at least would be more prone to developing αS pathology due to a neurological insult. These factors could provide a possible explanation for the differences in results observed in the two studies. The LPS used was derived from the same strain of *E. coli (E. coli* 0111:B4), and we injected twice the amount that Gao et al. injected (10ug versus 5ug), so the amount and type of LPS is most likely not responsible for the differences seen.

As LPS is known to invoke an inflammatory response, we looked for evidence of neuroinflammation in the mice. We saw the greatest number of astrocytes within the hippocampus and entorhinal cortex of αS injected mice, with cation exchanged αS and LPS injected mice also having elevated numbers of astrocytes compared to PBS injected mice. However, none of these reached statistical significance. This slight increase in astrogliosis provides a potential explanation to the reduced spread of αS pathology into the cortex of αS injected mice. The presence of LPS within the injected sample may be inducing an acute inflammatory response which could result in the removal of some of the αS seeds. This would lead to the reduction of spread of pathology, with the number of astrocytes returning to near-normal within the 3 months before the mice were sacrificed. Nevertheless, αS inclusions were present within astrocytes in both αS and cation exchanged αS fibril injected mice.

Serendipitously, we observed the presence of αS inclusions within ependymal cells in the αS (both αS and cation exchanged αS), but not in LPS or PBS injected M83 αS Tg mice. Furthermore, this ependymal αS inclusion pathology was not present in aged, motor impaired M83 αS Tg mice. Subsequent examination of M20 αS Tg mice injected with αS fibrils revealed a similar but reduced amount of αS pathology within the ependymal cells, and non-Tg mice injected with αS fibrils did not develop ependymal cell αS pathology. This suggests that ependymal αS inclusion pathology is specific to αS fibril injected αS Tg mice. Pathological studies of human α-synucleinopathies have described αS inclusions within astrocytes, oligodendrocytes, in the sub-ependymal area and between ependymal cells [45, 46].

The presence of αS inclusion pathology in ependymal cells provides a new level of complexity in interpreting the mechanism(s) involved in the spread of αS pathology, as it might provide additional explanations for the potent progression of this phenomenon in these mouse models. As ependymal cells line the ventricular system of the central nervous system, the uptake and release of αS aggregates from these cells into the cerebrospinal fluid would provide more widespread access to the brain and spinal cord, which could explain the efficient spread of αS inclusion pathology throughout the neuroaxis of these mice. Although some cell culture studies have provided decent evidence that αS conformational templating can occur under certain cellular conditions [47–49], the mechanisms involved in whole animal studies are more challenging to delineate due to the complex interactions of the biological mechanisms that can be involved [50, 51]. Given our new findings, additional studies are required to determine the relative contributions of various physiological and cellular mechanisms to the induction and spread of αS inclusion pathology in the αS fibril injected αS Tg mouse models.

## Conclusions

We have shown here that the presence of LPS in bacterially produced αS is not a major factor involved in the induction of αS inclusion pathology resulting from the cerebral injection of preformed recombinant αS amyloid seeds in M83 αS Tg mice. However, the presence of LPS levels should be considered when using bacterially expressed αS in biological studies. Furthermore we demonstrated the presence of αS inclusions within ependymal cells of αS Tg mice injected in the cerebrum with αS fibrils to induce the spread of αS inclusion pathology, which puts forth a novel mechanism that could contribute to this process in these animal models.

## Methods

### Antibodies

pSer129, also known as clone 81A, is a mouse monoclonal antibody that reacts with αS phosphorylated at Ser129 [52]. Syn506 is a conformational anti-αS mouse monoclonal antibody that preferentially detects αS in pathological inclusions [53, 54]. SNL4 is a rabbit polyclonal antibody that recognizes N-terminal residues 2–12 in αS [55]. Anti-p62 is a rabbit polyclonal antibody that is a general protein inclusion marker (SQSTM1; ProteinTech). Rabbit polyclonal anti-GFAP detects glial fibrillary acidic protein, and is a marker of astrocytes (Dako).

### Recombinant αS expression and purification

The bacterial expression plasmid; pRK172, encoding full length human αS was previously described [56]. Recombinant αS was expressed in BL21(DE3)/RIL *E. coli* (Agilent Technologies) and purified by size exclusion chromatography and subsequent Mono-Q ion exchange chromatography as previously described [56, 57]. To further purify αS and remove LPS contamination, we tested a number of different absorption resins including hydroxyapatite (Bio-Rad), hydrophobic resins, lipid removing agent (LRA; Sigma) and Mono-S ion exchange (Bio-Rad), using buffers with a range of pH values. In the most successful approach, samples were purified by High S Support cation exchange chromatography (Bio-Rad). The protein was exchanged with 20 mM PIPES, pH4.2, bound to the High S resin, extensively washed with 20 mM PIPES, pH4.2 and eluted with 20 mM PIPES, pH4.2, 1 M NaCl. The purified protein was then exchanged with PBS. Protein concentrations were quantified using the bicinchoninic acid (BCA) assay (Thermo Scientific), using bovine serum albumin as a standard (Pierce Biotechnology).

### Detection and quantification of endotoxin contamination

To assay for the presence of lipopolysaccharide (LPS) in the αS protein preparations, two methods were employed: 1) the HEK-Blue-hTLR4 cell culture system and QUANTI-Blue media (InvivoGen) according to the manufacturer’s protocol, and 2) the Pierce LAL Chromogenic Endotoxin Quantitation Kit (Thermo Scientific) according to the manufacturer’s protocol, using the included endotoxin standard for both assays. Standards and protein samples were prepared by dilution in endotoxin free water. Absorbance values were determined by plate reader, and for some samples were above the range of detection.

### Fibril formation of recombinant αS proteins

Fibrils were formed by incubating the αS proteins at 5 mg/ml in sterile PBS at 37 °C with constant shaking at 1050 rpm (Thermomixer R, Eppendorf) for 48 h. Fibril formation was monitored by (*trans*,*trans*)-1-bromo-2,5-bis-(4-hydroxy)styrylbenzene (K114) fluorometry as previously described [58]. Fibrils were prepared for injection by diluting the samples in sterile PBS followed by mild sonication in a water bath for 2 h.

### Mouse husbandry and stereotaxic brain injections

All procedures were performed according to the NIH Guide for the Care and Use of Experimental Animals and were approved by the University of Florida Institutional Animal Care and Use Committee. Tg mice expressing wild-type human αS (M20 line) or human αS containing the A53T mutation (M83 line), were previously described [40]. Two month old M83 αS Tg mice were bilaterally stereotaxically injected in the hippocampus (coordinates from Bregma: A/P −1.7, L ±1.6, D/V −2.0) with 2.5 μl αS fibrils (1.6 mg/mL) or αS fibrils purified of LPS (1.6 mg/ml), or 2.0 μl LPS (5 mg/ml; *E. coli* 0111:B4; Sigma) or sterile PBS (summarized in Table 1), at a rate of 0.2 μl/minute. A cohort of two month old M20 Tg mice and non-Tg mice was also stereotaxically injected in the hippocampus with 2.0 μl αS fibrils (2.0 mg/mL) or sterile PBS (M20 αS Tg mice only). Mice were sacrificed by CO_2_ euthanization followed by cardiac perfusion with PBS/heparin. The brain and spinal cord were harvested and fixed in 70 % ethanol/150 mM NaCl. The tissue was then dehydrated, paraffinized as previously described [59] and cut into 7 μm sections.

**Table 1:** Summary of αS Tg mice stereotaxically injected in the hippocampus

Mouse line	Number of mice	Months post-injection at death	Inoculate	Volume (μl)
M83^+/−^	4	3	αS (1.6 mg/ml)	2.5
M83^+/−^	4	3	Cation-exchanged αS (1.6 mg/ml)	2.5
M83^+/+^	4	2	LPS (5 mg/ml)	2
M83^+/+^	4	2	PBS	2
M20^+/-^	4	4	αS (2 mg/ml)	2
M20^+/-^	2	2	PBS	2
M20^+/-^	5	4	PBS	2
M20^+/-^	4	7	PBS	2
nTg	4	4	αS (2 mg/ml)	2

^+/−^ = hemizygous, ^+/+^ = homozygous

### Immunohistochemistry

Tissue sections were deparaffinized in xylene and rehydrated in a descending ethanol series (100 %, 90 %, 70 %). Antigen retrieval was performed by incubation in a steam bath for 30 min. Endogenous peroxidase activity was quenched, and samples were blocked with 2 % fetal bovine serum (FBS)/0.1 M Tris pH7.6. Primary antibodies were diluted in blocking solution and applied to samples for overnight incubation at 4 °C. Anti-rabbit or anti-mouse biotinylated antibodies were diluted in blocking solution and applied to sections for an hour at room temperature. Next, the avidin-biotin complex (ABC) Vectastain system (Vector Laboratories) was employed and immunocomplexes were visualized with the chromogen 3,3’-diaminobenzidine (DAB). Sections were counterstained with hematoxylin followed by dehydration in an ascending series of ethanols (70 %, 90 %, 100 %) and xylene. Sections were coverslipped using cytoseal and dried before scanning using an Aperio ScanScope CS (40x magnification; Aperio Technologies Inc.). Representative images (for figures) and images showing the hippocampus or the entorhinal cortex (for astrocyte counting) were taken using the ImageScope TM software (Aperio Technologies Inc.).

### Double immunofluorescence

Tissue sections were deparaffinized and rehydrated, and antigen retrieval was performed as described in the immunohistochemistry methods. Sections were blocked with 5 % dry milk/0.1 M Tris pH7.6. Primary antibodies were diluted in blocking solution and applied to sections for overnight incubation at 4 °C. Sections were washed with 0.1 M Tris pH7.6 and secondary antibodies conjugated to Alexa Fluor 488 or 594 fluorophores (Life Technologies) were diluted in blocking solution and applied to sections for 2 h at room temperature in the dark. Sections were then treated with sudan black to block lipofuscin autofluorescence. Nuclei were stained with 4’,6-diamidino-2-phenylindole (DAPI; Pierce) and sections were mounted using Fluoromount-G (SouthernBiotech). Pictures were obtained using an Olympus BX51 fluorescent microscope and images were overlaid using Photoshop CS6 software.

### Quantification of staining, cell counts and statistical analyses

Scanned images of Syn506 stained sections were loaded into ImageScope TM software. The hippocampal areas were outlined and analyzed for the abundance of DAB positive pixels. The burden was calculated by dividing the number of positive pixels by the area. Images obtained from the CA2/3 of the hippocampus and entorhinal cortex of GFAP-DAB stained sections were taken at 10x using ImageScope TM software. The images were randomized and coded, and the numbers of GFAP positive cells were counted by a single user, blinded to the experimental conditions. Two-tailed t-tests and one-way analysis of variance (ANOVA) with post-hoc Dunnett’s multiple comparison tests were performed in GraphPad Prism v5.03 software.

### Activation of an inflammatory response in primary microglia by purified LPS

The cortices from B6/C3H P0-P2 mice were isolated as previously described [60]. The mixed glial cultures were maintained in DMEM/10 % FBS with 100 units/ml penicillin and 100 μg/ml streptomycin. After 10–14 days of incubation, flasks were shaken at 150 rpm for 30 min at 37 °C to dislodge microglia from the astrocyte layer. The microglial cells were then re-plated into 6 well culture dishes in DMEM/10 % FBS with 100 units/ml penicillin and 100 μg/ml streptomycin. All cells were maintained at 37 °C in a humidified incubator with 5 % CO2. When the primary microglial cultures reached ~60 % confluency, the media was removed from the wells and was replaced with 2 ml of media containing 50 ng/ml LPS. Control wells received the same treatment, without the addition of LPS (*n* = 3/group). The cultures were returned to the incubator for 1, 6 or 12 h. Following incubation, images of the cultures were obtained using an EVOS FL cell imaging system (AMG). Then, the media was removed, mixed with protease inhibitors and aliquoted. The amount of IL-6 within the media (diluted 1:50) was determined using a BD OptEIA mouse IL-6 ELISA kit according to the manufacturer’s protocol (BD biosciences).

## Additional file

Additional file 1: Figure S1. Assessment of endotoxin activity within purified LPS. (A) Comparison of the activity of purified LPS (*E. coli* 0111:B4), that was used for cerebral injection, to standard endotoxin using the Pierce LAL assay. White bars represent the endotoxin standard and black bars represent test samples. (B) Images showing the morphology of primary microglia in culture following 6 and 12 h of treatment with 50 ng/ml purified LPS or nothing (control). Scale bar = 200 μm. (C) Detection of IL-6 within the media (diluted 1:50, 100 μl) taken from primary microglia cultures after 1 or 6 h of treatment with 50 ng/ml purified LPS or nothing (control). White bars represent IL-6 standard. Black bars represent test samples. Error bars represent the standard error of the mean. AU = absorbance units. (TIFF 4630 kb)

## Abbreviations

ABC: Avidin-biotin complex
ANOVA: Analysis of variance
αS: α-synuclein
BCA: Bicinchoninic acid
CNS: Central nervous system
CO_2_: Carbon dioxide
DAPI: 4’,6-diamidino-2-phenylindole
DAB: 3,3’-diaminobenzidine
DLB: Dementia with Lewy bodies
*E. coli*: *Escherichia coli*
EU: Endotoxin units
ELISA: Enzyme-linked immunosorbent assay
FBS: Fetal bovine serum
GFAP: Glial fibrillary acidic protein
HEK: Human embryonic kidney
IL-6: Interleukin-6
K114: (*trans*,*trans*)-1-bromo-2,5-bis-(4-hydroxy)styrylbenzene
LPS: Lipopolysaccharide
NAC: Non-amyloid component
PBS: Phosphate buffered saline
PD: Parkinson’s disease
Tg: Transgenic
TLR4: Toll-like receptor 4
