# Supplementary figures and images for: Studies of lipopolysaccharide effects on the induction of α-synuclein pathology by exogenous fibrils in transgenic mice
Source: Mol Neurodegener. 2015 Jul 30;10:32. doi: 10.1186/s13024-015-0029-4 (PMC4520273; doi:10.1186/s13024-015-0029-4)

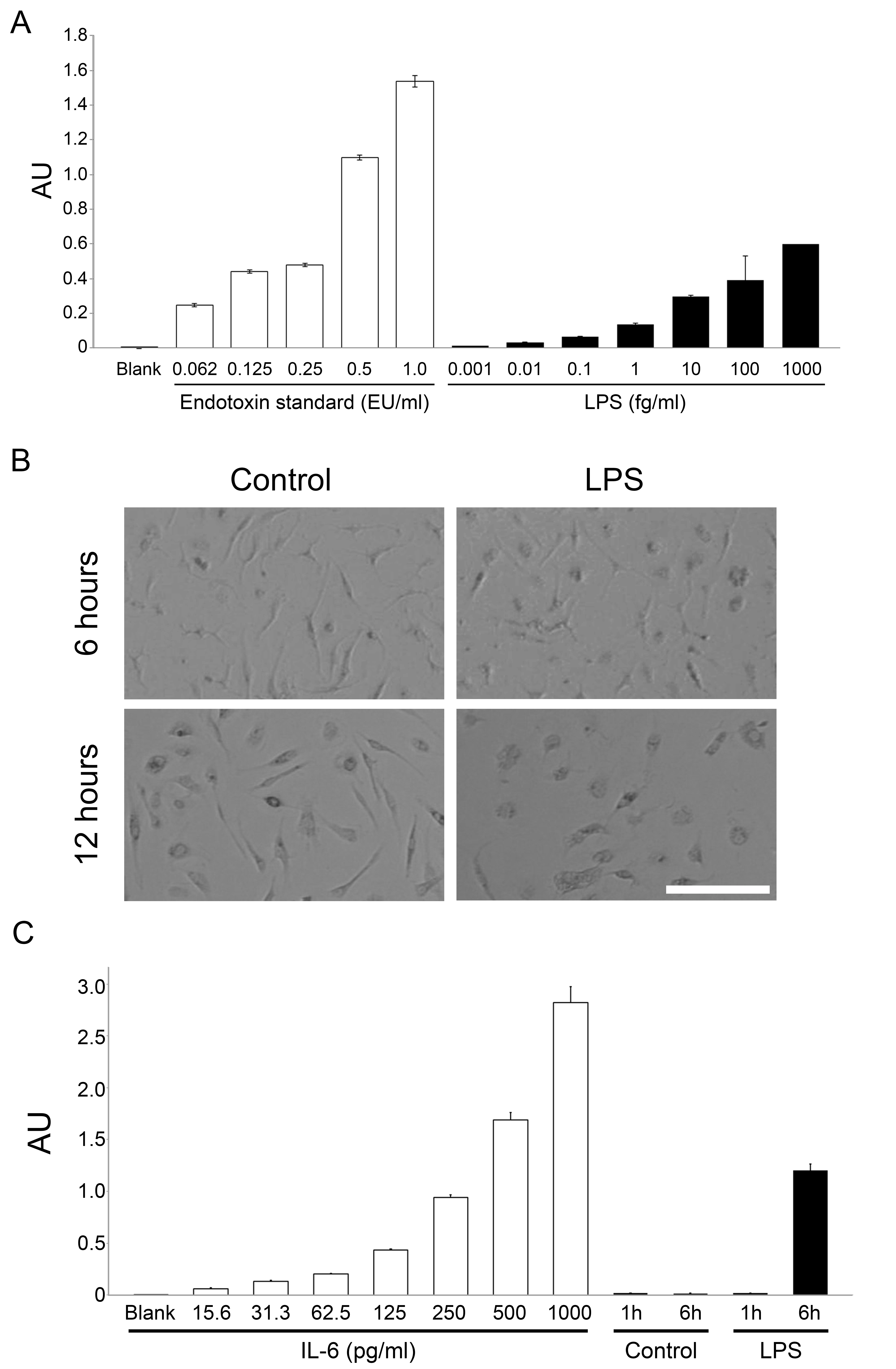

Supplement: Additional file 1: Figure S1. — Assessment of endotoxin activity within purified LPS. (A) Comparison of the activity of purified LPS (E. coli 0111:B4), that was used for cerebral injection, to standard endotoxin using the Pierce LAL assay. White bars represent the endotoxin standard and black bars represent test samples. (B) Images showing the morphology of primary microglia in culture following 6 and 12 h of treatment with 50 ng/ml purified LPS or nothing (control). Scale bar = 200 μm. (C) Detection of IL-6 within the media (diluted 1:50, 100 μl) taken from primary microglia cultures after 1 or 6 h of treatment with 50 ng/ml purified LPS or nothing (control). White bars represent IL-6 standard. Black bars represent test samples. Error bars represent the standard error of the mean. AU = absorbance units. (TIFF 4630 kb) [file 13024_2015_29_MOESM1_ESM.tif]
